# Inducing Error Management Culture – Evidence From Experimental Team Studies

**DOI:** 10.3389/fpsyg.2021.716915

**Published:** 2022-01-21

**Authors:** Alexander Klamar, Dorothee Horvath, Nina Keith, Michael Frese

**Affiliations:** ^1^Faculty of Humanities and Social Sciences, Work-, Organizational- and Business Psychology, Helmut-Schmidt-Universität/Universität der Bundeswehr Hamburg, Hamburg, Germany; ^2^Faculty of Business and Economics, Institute for Management and Organisation, Leuphana University of Lüneburg, Lüneburg, Germany; ^3^Department of Organizational and Business Psychology, Institute of Psychology, Technical University of Darmstadt, Darmstadt, Germany; ^4^Asia School of Business (in collaboration with MIT Sloan Management), Kuala Lumpur, Malaysia; ^5^Department of Management and Organization, NUS Business School, National University of Singapore, Singapore, Singapore

**Keywords:** errors, job and task performance, domain-specific culture, culture/climate change, culture/climate strength

## Abstract

Field studies indicate that error management culture can be beneficial for organizational performance. The question of whether and how error management culture can be induced remained unanswered. We conducted two experiments with newly formed teams, in which we aimed to induce error management culture and to explore whether we would also find beneficial effects of error management culture on performance in an experimental setting. Furthermore, we tested whether culture strength moderates the relationship between error management culture and performance. In Study 1, we used two tasks that require rational problem solving. In Study 2, we used a task that requires creative problem solving. We successfully manipulated error management culture in terms of an effect on perceived error management culture within the teams. While we did not find a direct effect of error management culture on performance, Study 2 revealed an indirect effect via communication in the teams. To our surprise, culture strength did not influence the hypothesized relationship. We discuss potential theoretical and alternative explanations for our results, and provide an outlook for future studies.

## Introduction, Theory, and Hypotheses Development

Errors occur every day, and in every organization. For individuals and organizations alike, it is thus of interest to learn how to deal with errors in order to be successful. *Error management* can be described as a perspective that pledges for a “useful approach to errors with the goal of reducing future errors, of avoiding negative error consequences and of dealing quickly with error consequences once they occur” (Frese, 1995, p. 113). We consider *errors* as unintentional deviations from a goal, rule, or standard (Reason et al., 1990; Frese and Zapf, 1994; Hofmann and Frese, 2011; Frese and Keith, 2015). It has to be noted that the error itself may be disentangled from its consequences. Negative error consequences may ultimately include *failure* (Frese and Keith, 2015). In an organizational context, failure refers to the “termination of an initiative to create organizational value that has fallen short of its goals” (Shepherd et al., 2011, p. 1229). Further, errors can be distinguished from *setbacks*. In a work environment, setbacks can be described as “task-related disruptions and inhibitions” (Chong et al., 2020, p. 1409). In that sense, a setback shares a similarity with an *inefficiency*: Given an inefficiency, the goal is ultimately reached. However, the path to reach the goal is not optimal, as it requires more time and/or resources. On the contrary, setbacks require action from the employees: When confronted with setbacks, employees have to “appraise unforeseen problems, unlearn their existing automatic task scripts promptly, develop novel solutions, learn new ways of operations, and adapt to updated rules and advisories” (Chong et al., 2020, p. 1410).

Error management acknowledges that despite best efforts to prevent errors, it is impossible to avoid errors completely (Reason, 1997). This suggests that dealing with errors after they have occurred is necessary. Error management elaborates on the aforementioned idea that errors can be distinguished from their consequences. Thus, errors do not inevitably lead to negative consequences; it is possible to avoid or reduce negative consequences, and even positive consequences can occur. Such positive consequences may be learning from errors (e.g., Sitkin, 1992). What is more, more learning occurs from failure than from success (Shepherd et al., 2011). Studies on error management have mostly focused on effectiveness of error management training for individuals, for example when principles of error management were incorporated in software training (e.g., Keith and Frese, 2008), or on processes and effects of error management on the individual level (e.g., Frese et al., 1991; Dormann and Frese, 1994; Chillarege et al., 2003; Heimbeck et al., 2003; Keith and Frese, 2005; Keith, 2011; for a meta-analysis, see Keith and Frese, 2008).

On a team and organizational level, team and organizational members may share a view on errors (e.g., they may consider errors as learning opportunities) and may have common practices in regard to errors (e.g., to openly discuss an error with colleagues). The norms and practices constitute an organizational culture (House et al., 2004). Organizational culture consists of the following components: First, norms are behavioral prescriptions that organizational members agree on. Second, these organizational norms are internalized by organizational members. Third, the normative ideas are reinforced by organizational members independent of supervisors or outside interventions. Fourth, through practice, the norms produce ‘normative pressure’ to conform (House et al., 2004).

*Error management culture^1^* (van Dyck et al., 2005) denotes one form of organizational culture with regard to errors. Given an error management culture, team members expect errors to happen – therefore, they are more vigilant and are better in anticipating errors. This allows them to detect errors faster. As a rule of thumb, the faster errors are detected, the better the chances to minimize negative error consequences (Keith and Frese, 2011). In field studies, error management culture has been shown to benefit organizational outcomes such as profitability, innovativeness, and safety (e.g., van Dyck et al., 2005; Hofmann and Mark, 2006; Keith and Frese, 2011; Fischer et al., 2018). While field studies have the advantage of high ecological validity, the higher ecological validity comes at the expense of lower internal validity, as external influences can hardly be excluded. This may be problematic, as many factors may play an important role and influence organizational performance, for example the leadership of the company, the industry in which the company operates, as well as other cultural factors that go above and beyond the error management culture. In different terms: While the results of these studies are quite impressive, the question remains whether the effect of error management culture on performance unfolds directly, or whether other variables may explain or modify this effect.

Most of the (field) studies on error management culture studied the effects of error management culture on the aggregated, organizational level instead of the more fine-grained team level (e.g., van Dyck et al., 2005; Fischer et al., 2018). Another shortcoming of these field studies is that the question of how an error management culture can be induced remained unanswered.

Inducing culture is not a trivial issue. There are several components that can go wrong if one wants to develop a (organizational) culture in a team: First, people may not agree on a norm. Therefore, the concept of ‘culture (or climate) strength’ has become important in the organizational culture literature (Schneider et al., 2002). If only a few unit members take the norm for granted, we cannot consider it culture or climate. In fact, one key element of culture is that it is shared and accepted by most (if not all) members of the unit (team, organization, society; e.g., House et al., 2004). Low culture strength of error management culture would thus mean a disagreement on how to consider errors and deal with occurring errors. Even if the mean value of perceived error management culture may be high, a disagreement would mean that not all team members in fact feel they may openly admit errors, or voice ideas without having to fear punishment for errors. For error management culture to truly *unfold*, both the mean level *and* culture strength have to be high. (If the mean level was low and culture strength high, this would indicate a culture where errors are considered negatively and have to be prevented.) Second, there may be superficial adjustments to instructions by supervisors (or, in an experimental setting, to the experimental instructions); thus, a certain internalization of the norms needs to take place (Gal’perin, 1967). Superficial adjustments to instructions may look very similar to norms, but should not be confused with culture, because people may merely repeat instructions (e.g., in a manipulation check) and this conformity may just reflect the willingness to participate in a study or the willingness to superficially conform to the supervisor. Third, culture needs to change behavior in the organizational unit: The first and certainly important change in behavior is related to communication behavior in the organizational unit (we further develop this issue in the following paragraph). However, communication behavior is just one prerequisite of organizational culture. Fourth, the willingness to conform to a certain cultural norm may be influenced by (a) the time that one spends practicing the norms in an organizational unit, (b) the adequacy of the organizational norm to the tasks that need to be done, and (c) the convincingness and obvious importance of the organizational norm. These issues have the following implications: First, we need to experiment with various instructions and methods of presenting the norms. Second, we also need to experiment with various tasks to find out which ones can be used for certain normative systems. And third, experimental approaches have inherent weaknesses because the time spent practicing the norms is usually highly limited.

Particularly in newly formed teams, communication may be influenced by error management culture, which may ultimately increase performance. We believe that this relationship is particularly important for tasks that require creative problem solving for the following reasons: First, a high error management culture may create an environment where people do not fear blame or punishment for erroneous ideas. Due to the lack of fear of reprisal, team members may dare to articulate ideas they would have kept to themselves in an environment where errors are punished. Furthermore, an error management culture can also be beneficial for resolving misunderstandings, as team members of teams high in error management culture may be more likely to actively ask questions and reassure themselves. A high error management culture fosters an atmosphere where communication about errors, exploration and experimentation, and thus the introduction of new ideas, processes or procedures are encouraged (Keith and Frese, 2011). In such a high error management culture, more communication – both formal and informal – should take place. In teams with a high error management culture, team members are more willing to approach others and ask for help when they cannot correct an error by themselves. Open communication can foster quick error detection and error handling (van Dyck et al., 2005). Moreover, error communication represents the most important practice of error management culture (van Dyck et al., 2005). Error communication denotes the tendency to openly discuss errors with others, without the fear of being punished. It can be assumed that sharing potentially harmful information – i.e., that one has made an error – will be reciprocated by the fellow team members. The reciprocation serves two purposes: for one, it reinforces the error management culture, where errors can be shared openly. For another, when error communication is well received by team members, the reciprocation reinforces future error communication. An open discussion of errors may thus foster communication in general.

Second, communication can foster performance: When more ideas are expressed, the final solution may be improved: “effective group processes, particularly those related to communication, increase information and so are essential for high-performing development processes” (Brown and Eisenhardt, 1995, p. 368). Particularly for complex tasks, exchanging ideas and collectively verifying applicability to the problem, the integration of different viewpoints may foster an augmented and common comprehension of the task at hand. This common understanding, combined with the joint pooling of ideas, may enhance performance. For the context of aviation, Foushee (1984) argues that “the process of interaction is related to group performance” (p. 273). Interaction may be particularly important for group performance when tasks require a deeper understanding of the matter, and where the solution is not pre-defined as one clear statement. Such tasks require at least some amount of creativity (West, 2000).

Research on virtual teams supports the importance of communication on team success (e.g., Piccoli et al., 2004; Cramton and Webber, 2005; Horwitz et al., 2006; Marlow et al., 2017; Eisenberg et al., 2019). Moreover, virtual teams exchange information less effectively than traditional teams (Hightower et al., 1998). As traditional team members spend a lot of time in the office together, they have many opportunities for informal communication, for example when taking lunchbreaks together, and maybe even when they meet privately after work. For project teams, the situation may be quite different: many project teams are comprised only for the duration of one specific project. When team members expect no further cooperation and interaction after the completion of the project, motivation and possibilities for informal interaction may be lower than in “regular” work teams. This may be problematic, as “what appear to be merely ‘casual conversations around the water cooler’ often serve to informally exchange the kinds of information and experience that are critical to project coordination. …. [These informal communication channels] help to fill in the details of work, handle exceptions, correct mistakes and bad predictions, and over time mange the ripple effects of previous decisions and actions” (Herbsleb and Grinter, 1999, p. 86). Furthermore, “since designs never exhibit perfect modularity and are never error-free, process execution is rarely flawless, and the world is never completely predictable, informal communication will be essential to maintain project coordination” (Herbsleb and Grinter, 1999, p. 94).

The aim of the present studies is thus (a) to explore how error management culture can be induced, (b) to investigate whether we can replicate the beneficial effect of error management culture on performance found in field studies under standardized conditions, as well as (c) to gain insights into potential variables modifying or explaining this relationship. To address these issues, we conducted two studies with newly formed teams, in which we aim to explore the following research question and hypotheses:

*Research Question 1*: How can error management culture be induced? (Studies 1 and 2).

*Hypothesis 1*: Error management culture positively predicts performance. (Studies 1 and 2).
*Hypothesis 2*: Culture strength moderates the relationship between error management culture and performance. (Studies 1 and 2).
*Hypothesis 3*: Communication mediates the effect between error management culture and performance. (Study 2).

In both studies, we follow an experimental approach and shift the focus from the organizational to the team level. By grouping strangers into teams – newly forming teams of people who have not known each other before – we have the opportunity to attempt to experimentally induce an error management culture.

We use an abductive approach (e.g., Bamberger, 2019) for our experiments in this field. We do not suggest that we will manage to achieve all aspects of the complex concept of organizational culture within two experiments. Rather, we think that we should get closer to an idea of how to develop an organizational culture of a team.

In Study 1, which we conducted in a laboratory setting, we employed two rational problem solving tasks, for which the degree of correctness varies gradually and can be objectively quantified. Employing two tasks enabled us to explore the duration of potential effects of our manipulation. In Study 2, which we conducted online, we employed one task that requires creative problem solving. We adapted our manipulations from Study 1 to fit the different task and context, and we additionally created a second, “slimmed,” manipulation for error management culture. The design of Study 2 (see below) further allowed us to analyze the amount of communication between the team members. Both rational and creative problem solving are important to attain organizational performance, and are adequate variables to study team behavior in an experimental, yet realistic context (in fact, participants in Study 2 believed to be working on an actual task for an actual company).

The crucial test whether culture has actually unfolded (as opposed to a mere compliance with instructions) examines the culture strength. According to the literature, culture strength should moderate the effects of error management culture on performance (Schneider et al., 2002).

It is highly relevant to study error management culture in an experimental setting, thereby allowing standardization and exclusion of other variables rather than statistically controlling for them. On the team level, different processes may be related to error management culture than to error management on the individual level. Our research contributes to the existing literature in the following ways. First, from an empirical perspective, we attempt to experimentally induce error management culture and to investigate the beneficial effects of error management culture on performance. Second, from a theoretical perspective, by studying how an error management culture can be induced, we aim to shed light on questions regarding the drivers of change and innovation effects. Third, we are among the first ones to explicitly take culture strength into consideration when studying effects of error management culture. Fourth, as field studies have repeatedly demonstrated beneficial effects of error management culture on performance, from a practitioner perspective, the question of how to induce an error management culture is highly relevant.

In the following, we describe the studies we conducted in detail, discuss potential explanations for our results, and provide an outlook for future studies.

## Study 1: Error Management Culture and Performance in Terms of Rational Problem Solving

### Method

#### Sample

Participants were 136 students (*N* = 44 triads and 2 dyads) of a mid-sized German university. Mean age was 22.14 years (*SD* = 3.20) and 69.1% were female. Most of the participants (67.7%) worked at least part-time. Participants received either EUR 8 (approximately USD 9.50) or partial course credit as compensation.

#### Experimental Design and Procedure

We invited participants into the laboratory in sessions of three persons each to work on two team tasks, namely, the “NASA Moon Survival Problem” task (Hall and Watson, 1970) and its variation “Survival at Sea.” In both tasks, participants had to rank 15 items of equipment in terms of their importance for survival. The tasks (and similar variations of it) are commonly used to study team decision making processes (e.g., Wanous and Youtz, 1986). For both tasks, expert solutions represent the optimal ranking of the items.

We asked participants to individually complete questionnaires regarding demographics and their attitudes about errors. Then, we grouped individual participants from the same session into teams of three and randomly assigned teams to one of two experimental conditions (between-participants design with one factor: error framing condition): (1) *Error management* or (2) *Error prevention*. In the *Error management* condition, participants were encouraged to make errors while working on the team task and to learn from them. In the *Error prevention* condition, participants were instructed to avoid errors while working on the team task. We asked participants to write down the most important points of the manipulations, formulated as action principles (Glaub et al., 2014) on a flipchart. Furthermore, we aimed at fostering internalization (e.g., Gal’perin, 1967) of the manipulations by repeating them several times throughout the experiment. After receiving the manipulations and the instructions for the first task (i.e., the NASA Moon Survival Problem), participants were asked to discuss what they had read about errors. Participants then had 20 min to work on the first task. After 20 min, participants were provided with the expert solution for the first task, and asked to calculate the difference of their solution to the expert solution. Participants were informed that these differences were considered as errors. Subsequently, participants individually had to complete questionnaires regarding how they perceived the work environment in the team. After completion of the questionnaires for the first task (see “Measures” section for details), participants received the second task (i.e., Survival at Sea). Again, participants were asked to commonly find a solution, then were provided with the expert solution, and lastly asked to complete questionnaires on the work environment in the team (see “Measures” section for details). Finally, participants were thanked, debriefed and compensated.

#### Measures

##### Perceived Error Management Culture

We assessed perceived error management culture after each task with the 17-item Error Management Culture Questionnaire (van Dyck et al., 2005; α = 0.90 after Task 1 and α = 0.91 after Task 2), with slight modifications of item wordings to fit the team context. For example, the original item “After making a mistake, people try to analyze what caused it” was changed to “After making a mistake, people in this team tried to analyze what had caused it.” The Error Management Culture Questionnaire (van Dyck et al., 2005) is commonly used as a measure in organizations (Frese and Keith, 2015). It entails aspects of error competence, learning from errors, analyzing errors, and error communication. Participants responded on a five-point Likert scale. Individual responses were aggregated at the team level. To justify aggregation, we computed within-team agreement for each team using *r*_wg(j)_ (James et al., 1984, 1993), and reliability of responses among team members with intraclass correlation coefficients (ICC; Bliese, 2000). The mean values of *r*_wg(j)_ = 0.84, ICC(1) = 0.28 and ICC(2) = 0.53 [*F*(45,90) = 2.13, *p* < 0.01] for Task 1 and *r*_wg(j)_ = 0.89, ICC(1) = 0.24 and ICC(2) = 0.48 [*F*(45,88) = 1.94, *p* < 0.01] for Task 2 suggested appropriate levels of within-team agreement and reliability (Le Breton and Senter, 2008).

In order to avoid potential confusions with our experimental intervention and independent variable error management framing, we will refer to our measure of error management culture as *perceived error management culture.*

##### Team Performance

As a measure of team performance, for each task, we calculated the difference between the team’s solution and the expert solutions (ranging from 0 to 112). We inverted the variable, so that higher values indicate smaller deviations from the expert solution, and thus better performance. In the following, we will refer to this as *closeness to the expert solution.*

##### Moderator Variable: Culture Strength

As a measure for culture strength (Schneider et al., 2002), we used *r*_wg(j)_-values of each team.

##### Control Variables

*Task Familiarity.* As task familiarity could influence performance in the task, we assessed task familiarity as a potential control variable. We asked participants whether they were familiar with the tasks, or had worked on the tasks before. A sample item is “Were you familiar with the ‘NASA Moon Survival Problem’ and the ‘Survival at Sea’ task?” Cronbach’s alpha was 0.79.

*Familiarity With Team Members.* As we recruited participants for Study 1 on campus, we considered the possibility that some participants may know one or both other team members. As we aimed to study how error management culture unfolds, and assumed that therefore, it was important to newly form teams, we decided to include familiarity with team members as control variable. We assessed if participants were familiar with their team members by asking “How well do you know the two other members of your group?” (from 1 = *not at all* to 5 = *very well*).

### Results

Means, standard deviations, and intercorrelations of Study 1 variables are provided in Tables 1, 2.

**TABLE 1:** Means, standard deviations, and intercorrelations of Study 1 variables.

Measure	M	SD	1	2	3	4	5	6	7
1. Manipulation (error framing condition)^a^									
Dependent variables									
2. (Team)performance at t1	75.94	10.04	0.10						
3. (Team)performance at t2	44.87	10.89	−0.18	0.05					
4. Perceived error management culture at t1	3.50	0.51	0.66**	–0.01	−0.21	(0.90)			
5. Perceived error management culture at t2	3.59	0.50	0.45**	–0.10	−0.02	0.80**	(0.91)		
Additional variables									
6. Task familiarity	1.92	0.15	0.15	0.03	−0.08	0.15	0.10	(0.79)	
7. Familiarity with other team members	1.78	0.98	0.02	0.09	−0.05	0.16	0.24	−0.27	-

*Cronbach’s alpha coefficients are shown in parentheses along the diagonal. N = 46 teams.*

*^a^Error framing condition was coded 0 for Error Prevention framing and 1 for Error Management framing.*

**p < 0.01.

**TABLE 2:** Means and standard deviations of dependent and process variables in Study 1 by between-participants factor levels.

		Error framing condition	
Measure		Error prevention	Error management
	*N*	23	23
Team performance at t1	*M*	74.87	77.01
	*SD*	10.98	9.11
Team performance at t2	*M*	46.78	42.96
	*SD*	10.73	10.95
Perceived error management culture at t1	M	3.16	3.84
	*SD*	0.49	0.24
Perceived error management culture at t2	M	3.37	3.82
	*SD*	0.53	0.37

To test whether we succeeded in inducing error management culture (Research Question 1) and if we can find the error management culture and performance link in teams (Hypothesis 1) for a rational problem solving task, we conducted mediation analyses (Preacher and Hayes, 2004), with error framing condition (i.e., Error Management or Error Prevention framing) as predictor variable, perceived error management culture as mediator, and performance (i.e., closeness to the expert solution) as criterion variable. We used 5,000 bootstrap samples and estimated 95% bootstrap confidence intervals (CIs). We included our control variables task familiarity and familiarity with team members in our analyses as covariates.

For the first task, we found that the error management culture manipulation led to a higher level of perceived error management culture for Task 1 than the error prevention culture manipulation, β = 1.28, *p* < 0.001. We did not find a relationship between perceived error management culture for Task 1 and performance, β = −0.18, *p* = 0.40. We did not find support for the indirect effect of error framing condition on team performance through perceived error management culture for Task 1, β = −0.23, CI [−0.83, 0.27] (see Table 3).

**TABLE 3:** Mediation analysis in Study 1.

	Path coefficients		Indirect effects	
	To error management culture at t1	To team performance at t1	Estimate	95% confidence interval
Manipulation (error framing)^1^	1.28 (0.12)**	0.43 (4.09)		
Error management culture at t1^2^		−0.18 (4.10)		
Manipulation → error management culture → team performance at t1			−0.23 (0.28)	–0.83, 0.27

	**To error management culture at t2**	**To team performance at t2**	**Estimate**	**95% confidence interval**

Manipulation (error framing)^1^	0.84 (0.13)**	−0.42 (3.71)		
Error management culture at t2^2^		0.11 (3.83)		
Manipulation → error management culture → team performance at t2			0.09 (0.17)	−0.23, 0.48

N = 46 teams, of which N_Error Prevention_ = 23 and N_Error Management_ = 23. Bootstrap confidence intervals were computed using 5,000 resamples. Total effect manipulation → team performance at t1 = 0.20 (3.07). Total effect manipulation → team performance at t2 = −0.33 (3.30). Standard errors in parentheses. **p < 0.001.

*^1^Error framing condition was coded 0 for Error Prevention framing and 1 for Error Management framing.*

*^2^As perceived by the team.*

For the second task, we found that the error management culture manipulation led to a higher level of perceived error management culture for Task 2 than the error prevention culture manipulation, β = 0.84, *p* < 0.001. We did not find a relationship between perceived error management culture for Task 2 and performance, β = 0.11, *p* = 0.55. We did not find support for the indirect effect of error framing condition on team performance through perceived error management culture for Task 2, β = 0.09, CI [−0.23, 0.48] (see Table 3).

While we succeeded in inducing error management culture in terms of an effect of our manipulation on perceived error management culture, we were not able to find a direct effect of error management culture on performance. This is contradictory to our expectations based on findings in field studies (e.g., van Dyck et al., 2005; Fischer et al., 2018). One possibility why we did not find the expected effect is that maybe we did not succeed in actually manipulating error management culture after all. It is possible that the effects we found on perceived error management culture are rather an indicator of superficial compliance with the instructions than an actual change in culture.

In order to test this hypothesis (Hypothesis 2), we decided to take a closer look at culture strength (i.e., the agreement about the groups’ culture between team members; Schneider et al., 2002) regarding error management culture. In order to explore whether error management culture predicted performance in teams with a high culture strength, we tested whether culture strength moderates the relationship between perceived error management culture and performance (Hypothesis 2).

For Task 1, we conducted a moderation analysis using multiple linear regression, with perceived error management culture in Task 1 as predictor, culture strength in terms of *r*_wg(j)_ as moderator, and performance in Task 1 as criterion variable. We did not find a significant main effect of perceived error management culture (β = 0.24, *p* = 0.81), nor of culture strength (β = 0.60, *p* = 0.72). Further, we did not find a significant interaction effect of perceived error management culture and culture strength (β = −0.67, *p* = 0.77) (*R*^2^ = 0.01; *F* = 0.16, *p* = 0.92).

Similarly, for Task 2, we conducted a moderation analysis using multiple linear regression, with perceived error management culture in Task 2 as predictor, culture strength in terms of *r*_wg(j)_ as moderator, and performance in Task 2 as criterion variable. We did not find a significant main effect of perceived error management culture (β = 0.87, *p* = 0.69), nor of culture strength (β = 0.41, *p* = 0.87). Further, we did not find a significant interaction effect of perceived error management culture and culture strength (β = −1.28, *p* = 0.74) (*R*^2^ = 0.13; *F* = 2.01, *p* = 0.13). Thus, Hypothesis 2 is rejected (see Table 4).

**TABLE 4:** Moderation analysis for culture strength in Study 1.

Criterion variable	Predictor	B	SE(B)	β	t	p
Team performance at t1^1^	Error management culture at t1^2^	4.69	19.09	0.24	0.25	0.81
	Culture strength at t1	22.30	61.80	0.60	0.36	0.72
	Error management culture at t1 × culture strength at t1	–6.18	20.61	–0.67	–0.30	0.77
Team performance at t1^3^	Error management culture at t2	18.82	46.22	0.87	0.41	0.69
	Culture strength at t2	26.77	156.17	0.41	0.17	0.86
	Error management culture at t1 × culture strength at t2	–16.79	49.92	–1.29	–0.34	0.74

*N = 46 teams, of which N_Error Prevention_ = 23 and N_Error Management_ = 23.*

*^1^R^2^ = 0.01.*

*^2^As perceived by the team.*

*^3^R^2^ = 0.13.*

### Discussion

In Study 1, we were successful in manipulating error management culture in terms of an effect of our manipulation on perceived error management culture (Research Question 1). However, we could not find a beneficial effect of error management culture on performance in terms of rational problem solving (Hypothesis 1). Our additional analysis did not provide evidence for our speculation of a moderating effect of culture strength on the effect of perceived error management culture on performance. In concrete terms, we did not find evidence that perceived error management culture was beneficial for performance in teams with high culture strength, but not for teams with low culture strength.

We assumed that one of the reasons we did not find an effect of error management culture may lie in the type of tasks we had used – tasks that required rational problem solving. In order to effectively work on tasks that require rational problem solving, more analytic, convergent thinking may be required. Teams, particularly newly formed teams, that work on tasks that require rational problem solving may discuss in a focused, goal-oriented way (Guilford, 1957), and try to avoid errors whenever possible. On the contrary, in tasks that require creative problem solving, such as brainstorming, divergent thinking may be an effective strategy. For effectively conducting brainstorming tasks, it is particularly important that team members voice their ideas, without any limitations or barriers as to whether the idea may be implemented. Open communication is a vital part of error management culture. Therefore, we assumed that error management culture may be particularly beneficial for tasks that require creative problem solving, such as brainstorming. We aimed to address these possibilities in Study 2. As in Study 1, the pattern of results was the same for both tasks, for Study 2, we decided to employ only one task.

## Study 2: Error Management Culture and Performance in Terms of Creative Problem Solving

We conducted Study 2 as an online experiment with newly formed teams. In Study 2, participants’ task was to create a marketing plan for a certain product (Hubner et al., 2020). While there are some factors that need to be considered when creating a marketing plan (such as who is the target group, how to advertise for the product, where to advertise, etc.), it is a task that requires a considerable amount of creativity. Similar to a brainstorming task, at first, one may collect ideas, and only in a second phase, the ideas are evaluated and selected, before agreeing on a common marketing plan.

We formed teams by grouping individual participants who had not known each other, and we attempted to manipulate error management culture. We had planned to comprise teams of three participants. However, as we had observed that some participants dropped out before the other two team members had shown up, we also used data of dyads, but included team size as control variable.

In Study 2, we employed the same manipulations as in Study 1 (after adapting them to fit the task and context). We additionally created a “slimmed” manipulation for error management culture. The main difference of both error management culture manipulations was that in the “slimmed” version, we did not ask participants to develop, formulate and note action principles for error management. Thereby, we aimed to address the issue that in the online environment, we were not able to control whether participants actually complied with our manipulation and did, in fact, formulate action principles. Different effects for both manipulations may thus suggest that participants in the “regular” error management condition actually complied with the manipulation and formulated action principles, and that these action principles were an important aspect of the manipulation.

### Method

#### Sample

Participants were working adults from the United States recruited online using Amazon’s Mechanical Turk. Previous research has shown that data gathered from such environments is of acceptable quality (e.g., Buhrmester et al., 2011). We carefully followed specific suggestions that shall help to further enhance this quality: We used attention check items (e.g., “I receive my paycheck from goblins”; Meade and Craig, 2012), a manipulation check that measures understanding of instructions (see below) as a prerequisite to further participate in the experiment, as well as fair compensation (e.g., Aguinis and Lawal, 2012; Cheung et al., 2017). Additionally, in order to statistically control for potentially lower commitment to the participation in the study, we assessed and controlled for goal commitment (see below). The criteria for inclusion of respondents in the survey were age (>18 years) and place of residence (United States). Fourteen participants did not meet our criteria for inclusion and were excluded from further analyses. The final sample consisted of 309 participants (*N_total_* = 128 teams, of which *n_triads_* = 53 and *n_dyads_* = 75). Mean age of participants was 35.81 years (*SD* = 11.53) and 43.9% were female^2^. Participants’ average work-experience was 13.00 years (*SD* = 10.08) and 32.5% reported to hold a leadership position. Participants received USD 4.50 for participation (which corresponds to an hourly wage of approximately USD 9 and was thus in line with the United States federal minimum wage).

#### Experimental Design and Procedure

Participants were invited to work on a team task, namely, to develop a marketing plan for a newly developed product (Hubner et al., 2020). The product was fictitious, but as Hubner et al. (2020) demonstrated, participants deem it as realistic and often work enthusiastically on this task. The instructions explained that a start-up is currently working on the marketing concept of its most promising product, but is not sure how to advertise for it. Therefore, the start-up asks the “wisdom of the crowd” for help. Participants were explained that their task is embedded in a research project and that they also have to complete a questionnaire after working on the team task.

Participants were randomly assigned to one of three experimental conditions (between-participants design, one factor error framing condition with three levels): (1) a “slimmed” *Error Management framing* condition that did not foster internalization, (2) an *Error Management framing* condition similar to that of Study 1, and (3) an *Error Prevention framing* condition similar to that of Study 1. Participants who were grouped together received the same manipulation. In order to keep the manipulation realistic and in line with what is common in online environments such as Amazon’s Mechanical Turk, the manipulation was part of the written instructions that participants received for task completion. Our manipulations focused on how participants should deal with errors in the process of the team discussion. In the slimmed *Error Management framing* manipulation, we encouraged participants to make errors and to learn from them. In the *Error Management framing* condition similar to that of Study 1, we additionally aimed to foster internalization of the manipulations (e.g., Gal’perin, 1967). For this purpose, we asked participants to write down two error principles on a sheet of paper so they should be able to see them the entire time when working on the team task: “errors are positive” and “talk about errors openly in the team, as you can learn from them.” These principles should serve as guidelines to follow while working on the team task. In the *Error Prevention framing* condition similar to that of Study 1, participants were instructed to avoid errors. We also asked them to follow two error principles: “avoid errors as they only bother you and slow you down” and “make the marketing plan as perfect as possible” right from the start. We also asked participants to write down the error principles on a sheet of paper so they should be able to see them the entire time when working on the team task.

After reading the instructions, participants were asked to respond to questions assessing the understanding of the instructions. Participants who had received the same manipulation (i.e., either *“slimmed” Error Management, Error Management* or *Error Prevention framing*) and answered the questions correctly then arrived on a page containing a built-in chatroom. After the team members arrived on the page with the chatroom, the chat-function was automatically enabled. Participants could start to chat and generate ideas for a marketing plan. The teams had a maximum of 20 min time to complete the task. The chat window was programmed to close automatically 30 min after the first participant had logged in. Three minutes prior to that, participants were informed about the remaining time. After the chatroom closed, participants had to submit the team’s common final ideas on the next page. Subsequently, participants individually had to fill out questionnaires regarding how they perceived the work environment in the team (see “Measures” section for details). Finally, participants were thanked, debriefed and provided with a code for payment on Amazon’s Mechanical Turk.

#### Measures

##### Understanding of Instructions

After reading the instructions and before the dependent variable was assessed, participants responded to questions that probed whether participants had understood what the task was about and what the instructions had stated about errors. We asked participants three questions (“What does our most promising product do?,” “What task do you have to accomplish in this project?,” and “What was written in the text about making errors while working in a team?”) and they had to choose the correct answer out of four possibilities. A false answer led to an exclusion from the study, as it indicated that participants had not read the instructions carefully.

##### Error Management Culture

As in Study 1, we assessed error management culture with the 17-item Error Management Culture Questionnaire (van Dyck et al., 2005; α = 0.94), with slight modifications of item wordings to fit the team context. Participants responded on a five-point Likert scale. Individual responses were aggregated at the team level. To assess whether aggregation is justified, we computed within-team agreement for each team using *r*_wg(j)_ (James et al., 1984, 1993), and reliability of responses among team members with intraclass correlation coefficients (ICC; Bliese, 2000). The mean values of *r*_wg(j)_ = 0.83, ICC(1) = 0.15 and ICC(2) = 0.30 [*F*(127,177) = 1.43, *p* < 0.05] suggested low but still appropriate levels of within-team agreement and reliability (Le Breton and Senter, 2008), justifying aggregation.

##### Communication

We assumed that teams that communicated more with each other during the brainstorming task may produce more creative ideas. We thus observed the communication of the teams in terms of the number of words exchanged during the group discussion. We analyzed the chat protocols and counted the number of words that the participants exchanged while working on the task. As this is an objective measure, within-team consistency and agreement measures are not applicable.

##### Dependent Variables

We used two dependent variables as indicators of performance: the quality of the ideas for the marketing plan, and the quantity of the ideas the marketing plan consisted of. We operationalized *quality of the ideas* by assessing three characteristics of the marketing plans: originality, usefulness, and completeness (Dean et al., 2005). Dean et al. (2005) defined an idea as original when it “rare (…), ingenious, imaginative, or surprising” (p. 663). To assess usefulness, we oriented on Dean et al.’s (2005) definition of workability/feasibility: the idea has to be “easily implemented and does not violate known constraints” (Dean et al., 2005, p. 663). An idea can be considered as complete when it covers aspects such as “who, what, where, when, why, and how” (Dean et al., 2005, p. 663). Two independent raters who were blind to the conditions rated the marketing plans on originality, usefulness, and completeness (all ICCs > 0.70). Subsequently, originality, usefulness, and completeness (α = 0.87) were combined as a measure for the quality of the ideas for the marketing plan. We operationalized *quantity of the ideas* as the number of ideas submitted for the marketing plan. For this purpose, we counted the number of discrete ideas submitted by the teams.

##### Moderator Variable: Culture Strength

As in Study 1, we used *r*_wg(j)_-values of each team as a measure for culture strength (Schneider et al., 2002).

##### Control Variables

*Team Size.* We controlled for *team size* because the amount of communication in the group may be higher in teams of three members than in teams of two members.

*Task Familiarity.* We included *task familiarity* as control variable, because both the quality and the quantity of ideas for the marketing plan may depend on how familiar participants are with similar tasks. We assessed task familiarity with the following three questions: “How familiar are you with creativity tasks, such as the task you were working on?”; “How familiar are you with creativity methods, such as brainstorming?”; “How experienced are you with marketing (from work, university, etc.)?” Participants responded on a five-point Likert scale. Cronbach’s alpha was 0.82.

*Goal Commitment.* Particularly for studies that are conducted online, the extent to which participants take the task seriously may influence the results. We therefore included *goal commitment* as control variable. We assessed goal commitment with three items of Hollenbeck et al.’s (1989) nine-item Goal Commitment scale. One sample item is “I was strongly committed to pursuing our goal of submitting a marketing plan.” Participants responded on a five-point Likert scale. Cronbach’s alpha was 0.72.

### Results

Means, standard deviations, and correlations of Study 2 variables are provided in Tables 5, 6.

**TABLE 5:** Means, standard deviations, and intercorrelations of Study 2 variables.

	M	SD	1	2	3	4	5	6	7	8	9	10
**Manipulation**												
1. Error management framing vs. others^a^	−	−	−									
2. “Slimmed” error management framing vs. others^b^	−	−	−0.47**	−								
3. Error prevention framing vs. others^c^	−	−	−0.53**	−0.50**	−							
**Dependent variable (team) performance**												
4. Quality of ideas	2.90	0.52	0.04	0.13	–0.16	(0.87)						
5. Quantity of ideas	3.90	1.73	–0.09	0.19*	–0.09	0.62**	−					
Process variables												
6. Perceived error management culture	3.74	0.59	0.12	–0.04	–0.09	0.27**	0.19*	(0.94)				
7. Communication	502.67	285.64	0.06	0.09	–0.15	0.44**	0.49**	0.25**	−			
**Control variables**												
8. Team size^d^	−	−	0.07	0.06	–0.13	0.19*	0.26**	0.18*	0.42**	−		
9. Task familiarity	2.76	0.66	–0.15	0.13	0.03	0.06	–0.02	0.25**	−0.24**	–0.08	(0.82)	
10. Goal commitment	4.57	0.46	–0.12	0.04	0.08	0.28**	0.18*	0.38**	0.16	0.19*	0.15	(0.72)

*Cronbach’s alpha coefficients are shown in parentheses along the diagonal. N = 128 teams.*

*^a^Error management framing vs. others was coded −1 for Error prevention framing, −1 for “Slimmed” error management framing, and 2 for Error management framing.*

*^b^“Slimmed” error management framing vs. others was coded −1 for Error prevention framing, 2 for “Slimmed” error management framing, and −1 for Error management framing.*

*^c^Error prevention framing vs. others was coded 2 for Error prevention framing, −1 for “Slimmed” error management framing, and −1 for Error management framing.*

*^d^Team size was coded 0 = two team members, and 1 = three team members.*

*p < 0.05, **p < 0.01.

**TABLE 6:** Means and standard deviations of dependent and process variables in Study 2 by between-participants factor levels.

		Error framing condition								
		Error prevention			Error management			“Slimmed” error management		
Measure		Total	Dyads	Triads	Total	Dyads	Triads	Total	Dyads	Triads
	*N*	46	31	15	43	23	20	39	21	18
Quality of the ideas	*M*	2.79	2.73	2.92	2.93	2.96	2.90	3.00	2.80	3.23
	*SD*	0.49	0.50	0.44	0.53	0.53	0.56	0.52	0.49	0.46
Quantity of the ideas	*M*	3.70	3.68	3.73	3.67	3.43	3.95	4.38	3.38	5.56
	*SD*	1.47	1.64	1.10	1.66	1.78	1.50	2.02	1.66	1.79
Perceived error management culture	*M*	3.68	3.69	3.65	3.85	3.71	4.00	3.71	3.55	3.90
	*SD*	0.68	0.73	0.57	0.56	0.56	0.52	0.50	0.47	0.48
Communication	*M*	446.52	364.94	615.13	527.09	420.74	649.40	541.97	434.10	667.83
	*SD*	245.57	204.79	242.43	348.72	233.50	419.81	246.54	178.61	259.21

*N = 128 teams.*

To test whether we succeeded in inducing error management culture (Research Question 1) and whether we could find an effect of perceived error management culture on performance (Hypothesis 1), we conducted mediation analyses (Preacher and Hayes, 2004; Hayes and Preacher, 2014) with error framing condition (i.e., Error Management vs. Error Prevention vs. “slimmed” Error Management framing) as predictor variable, perceived error management culture as mediator, and performance (in terms of quality and quantity) as criterion variables. As in Study 1, we used 5,000 bootstrap samples and estimated 95% bootstrap CIs, and we included team size, task familiarity, and goal commitment as covariates. For our multicategorical predictor variable (i.e., error framing condition), we created two dummy variables with indicator coding and the Error Prevention framing as reference category: D1 with codes of (0, 1, 0) for Error Prevention framing, Error Management framing, and “slimmed” Error Management framing, respectively, and D2 with codes of (0, 0, 1) for Error Prevention framing, Error Management framing, and “slimmed” Error Management framing, respectively.

**FIGURE 1:**
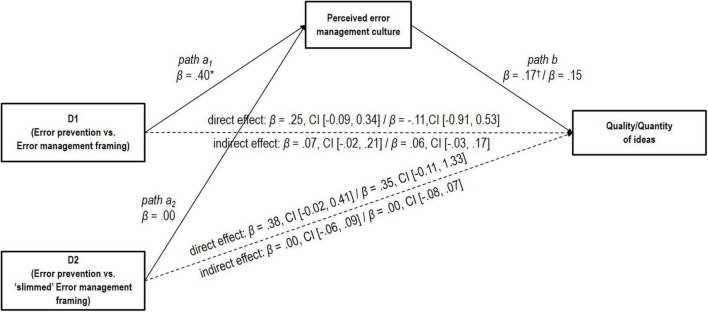
Original mediation model in Study 2. The non-significant indirect effect of dummy variable D1 for our manipulation [i.e., error prevention (coded 0) vs. error management framing (coded 1)] and the non-significant indirect effect of dummy variable D2 for our manipulation [i.e., error prevention (coded 0) vs. “slimmed” error management framing (coded 1)] on performance (quality or quantity of ideas) through perceived error management culture in Study 2. The dashed arrows indicate the direct paths between the dummy variables for our manipulation (D1 and D2) and performance. Standardized and partially standardized values and confidence intervals (CI). *N* = 128 teams, ^†^*p* < 0.10, *p < 0.05.

We found that the Error Management framing manipulation led to a higher level of perceived error management culture than the Error Prevention framing manipulation, D1: β = 0.40, *p* < 0.05 (see Figure 1 path a_1_). The “slimmed” Error Management framing manipulation did not lead to a higher level of perceived error management culture than the Error Prevention framing manipulation, D2: β = 0.00, *p* = 0.99 (see Figure 1 path a_2_). As in Study 1, we could not find the relationship between perceived error management culture and performance, β = 0.17, *p* = 0.08 for quality of the ideas (see Figure 1 path b), and β = 0.15, *p* = 0.13 for quantity of the ideas (see Figure 1 path b). We did not find support for the indirect effect of the experimental manipulation on performance (neither in terms of quality nor quantity of the ideas) through perceived error management culture, neither for our first dummy variable (D1) “Error Prevention framing vs. Error Management framing,” β = 0.07, CI [−0.02, 0.21] for quality of the ideas, and β = 0.06, CI [−0.03, 0.17] for quantity of the ideas (see Figure 1), nor for our second dummy variable (D2) “Error Prevention framing vs. “slimmed” Error Management framing” β = 0.00, CI [−0.06, 0.09] for quality of the ideas, and β = 0.00, CI [−0.08, 0.07] for quantity of the ideas (see Table 7 and Figure 1).

**TABLE 7:** Mediation analysis in Study 2.

	Path coefficients		Indirect effects	
	To error management culture	To team performance in terms of quality	Estimate	95% confidence interval
Manipulation X1^1^	0.40 (0.11)*	0.25 (0.11)		
Manipulation X2^2^	0.00 (0.12)	0.38 (0.11)		
Error management culture^3^		0.17 (0.08)		
Manipulation X1 → error management culture → team performance in terms of quality			0.07 (0.06)	−0.02, 0.21
Manipulation X2 → error management culture → team performance in terms of quality			0.00 (0.04)	−0.06, 0.09

	**To error management culture**	**To team performance in terms of quantity**	**Estimate**	**95% confidence interval**

Manipulation X1	0.40 (0.11)*	−0.11 (0.36)		
Manipulation X2	−0.00 (0.12)	0.35 (0.36)		
Error management culture		0.15 (0.29)		
Manipulation X1 → error management culture → team performance in terms of quantity			0.06 (0.05)	−0.03, 0.17
Manipulation X2 → error management culture → team performance in terms of quantity			0.00 (0.03)	−0.08, 0.07

*N = 128 teams, of which N_Error Prevention_ = 46, N_Error Management_ = 43, N_“Slimmed”Error Management_ = 39. Bootstrap confidence intervals were computed using 5,000 resamples. Total effect manipulation X1 → team performance in terms of quality = 0.31 (0.11). Total effect manipulation X2 → team performance in terms of quality = 0.38 (0.11). Total effect manipulation X1 → team performance in terms of quantity = −0.05 (0.36). Total effect manipulation X2 → team performance in terms of quantity = 0.35 (0.37). Standard errors in parentheses.*

*p < 0.05.

*^1^X1 was coded 0 for Error Prevention framing and 1 for Error Management framing.*

*^2^X2 was coded 0 for Error Prevention framing and 1 for slimmed Error Management framing.*

*^3^As perceived by the team.*

To test whether culture strength moderated the relationship of error management culture and performance (Hypothesis 2), we conducted a moderation analysis using multiple linear regression, with perceived error management culture as predictor, culture strength in terms of *r*_wg(j)_ as moderator, and performance (both in terms of quality and quantity of the ideas) as criterion variable. For performance in terms of quality of the ideas, we did not find a significant main effect of perceived error management culture (β = 0.02, *p* = 0.92), nor of culture strength (β = −0.96, *p* = 0.26). Further, we did not find a significant interaction effect of perceived error management culture and culture strength (β = 0.26, *p* = 0.30) (*R*^2^ = 0.06; *F* = 2.74, *p* = 0.05). For performance in terms of quantity of the ideas, we did not find a significant main effect of perceived error management culture (β = 0.32, *p* = 0.68), nor of culture strength (β = −1.63, *p* = 0.58). Further, we did not find a significant interaction effect of perceived error management culture and culture strength (β = 0.44, *p* = 0.61) (*R*^2^ = 0.04; *F* = 1.88, *p* = 0.14) either. Thus, Hypothesis 2 is rejected (see Table 8).

**TABLE 8:** Moderation analysis for culture strength in Study 2.

Criterion variable	Predictor	B	SE(B)	β	t	p
Team performance (in terms of quality of the ideas)^1^	Error management culture^2^	0.02	0.22	0.02	0.09	0.92
	Culture strength	–0.96	0.86	–0.58	–1.12	0.26
	Error management culture × culture strength	0.26	0.25	0.66	1.04	0.30
Team performance (in terms of quantity of the ideas)^3^	Error management culture	0.32	0.77	0.10	0.42	0.68
	Culture strength	–1.64	2.95	–0.29	–0.55	0.58
	Error management culture × culture strength	0.44	0.87	0.33	0.51	0.61

*N = 128 teams, of which N_Error Prevention_ = 46, N_Error Management_ = 43, N_“Slimmed”Error Management_ = 39.*

*^1^R^2^ = 0.06.*

*^2^As perceived by the team.*

*^3^R^2^ = 0.04.*

**FIGURE 2:**
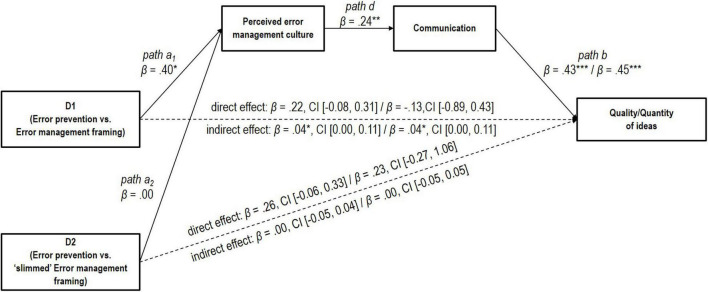
Alternative mediation model in Study 2. The significant indirect effect of dummy variable D1 for our manipulation [i.e., error prevention (coded 0) vs. error management framing (coded 1)] and the non-significant indirect effect of dummy variable D2 for our manipulation [i.e., error prevention (coded 0) vs. “slimmed” error management framing (coded 1)] on performance (quality or quantity of ideas) through perceived error management culture and communication in Study 2. The dashed arrows indicate the direct paths between the dummy variables for our manipulation (D1 and D2) and performance. Standardized and partially values and confidence intervals (CI). *N* = 128 teams, *p < 0.05, **p < 0.01, ***p < 0.001.

To test whether perceived error management culture affected performance indirectly through communication (Hypothesis 3), we conducted serial mediation analyses (Preacher and Hayes, 2004; Hayes and Preacher, 2014) with error framing condition as predictor, perceived error management culture and communication as mediators, and performance (in terms of quality of the ideas or quantity of the ideas) as criterion variables. We used 5,000 bootstrap samples and estimated 95% bootstrap CIs. We found that the Error Management framing manipulation led to a higher level of perceived error management culture than the Error Prevention framing manipulation, D1: β = 0.40, *p* < 0.05 (see Figure 2 path a_1_). The “slimmed” Error Management framing manipulation did not lead to a higher level of perceived error management culture than the Error Prevention framing manipulation, D2: β = 0.00, *p* = 0.99 (see Figure 2 path a_2_). Furthermore, perceived error management culture positively predicted communication, β = 0.24, *p* < 0.01 (see Figure 2 path d), and communication positively predicted performance both in terms of quality of the ideas, β = 0.43, *p* < 0.001 (see Figure 2 path b), and quantity of the ideas, β = 0.45, *p* < 0.001 (see Figure 2 path b). The 95% bias corrected confidence interval for the indirect effect excluded zero, indicating a significant indirect relationship for our first dummy variable (D1) “Error Prevention framing vs. Error Management framing” with performance (both in terms of quality of the ideas and quantity of the ideas), β = 0.04, CI [0.00, 0.11] for quality of the ideas, and β = 0.04, CI [0.00, 0.11] for quantity of the ideas (see Figure 2). In other words, the results are consistent with the idea that perceived error management culture and communication mediate the relationship between error framing condition and performance. For our second dummy variable (D2), “Error Prevention framing vs. ‘slimmed’ Error Management framing,” we did not find an indirect relationship with performance (in terms of quality of the ideas or quantity of the ideas), β = 0.00, CI [−0.05, 0.04] for quality of the ideas, and β = 0.00, CI [−0.05, 0.05] for quantity of the ideas (see Table 9 and Figure 2).

**TABLE 9:** Serial mediation analysis with communication in Study 2.

	Path coefficients			Indirect effects	
	To error management culture	To communication	To team performance in terms of quality	Estimate	95% confidence interval
Manipulation X1^1^	0.40 (0.11)*	0.05 (54.10)	0.22 (0.10)		
Manipulation X2^2^	0.00 (0.12)	0.27 (54.10)	0.36 (0.10)		
Error management culture^3^		0.24 (42.44)**	0.06 (0.08)		
Communication			0.43 (0.00)***		
Manipulation X1 → Error management culture → Communication → Team performance (quality)				0.04 (0.03)*	0.00, 0.11
Manipulation X2 → error management culture → communication → team performance (quality)				0.00 (0.02)	−0.05, 0.04

	**To error management culture**	**To communication**	**To team performance in terms of quantity**	**Estimate**	**95% confidence interval**

Manipulation X1^1^	0.40 (0.11)*	0.05 (54.10)	−0.13 (0.33)		
Manipulation X2^2^	0.00 (0.12)	0.27 (54.10)	0.23 (0.34)		
Error management culture^3^		0.24 (42.44)*	0.04 (0.27)		
Communication			0.45 (0.00)*		
Manipulation X1 → Error management culture → Communication → Team performance (quantity)				0.04 (0.03)*	0.00, 0.11
Manipulation X2 → Error Management culture → Communication → Team performance (quantity				0.00 (0.02)	−0.05, 0.05

*N = 128 teams, of which N_Error Prevention_ = 46, N_Error Management_ = 43, N_“Slimmed”Error Management_ = 39. Bootstrap confidence intervals were computed using 5,000 resamples. Total effect manipulation X1 → team performance in terms of quality = 0.31 (0.11). Total effect manipulation X2 → team performance in terms of quality = 0.38 (0.11). Total effect manipulation X1 → team performance in terms of quantity = −0.05 (0.36). Total effect manipulation X2 → team performance in terms of quantity = 0.35 (0.37). Standard errors in parentheses.*

*p < 0.05; **p < 0.01; ***p < 0.001.

*^1^X1 was coded 0 for Error Prevention framing and 1 for Error Management framing.*

*^2^X2 was coded 0 for Error Prevention framing and 1 for slimmed Error Management framing.*

*^3^As perceived by the team.*

### Discussion

In sum, regarding Research Question 1, we succeeded in inducing error management culture in terms of an effect on perceived error management culture using the same type of manipulation as in Study 1, i.e., our manipulation containing action principles. Our “slimmed” manipulation (that did not ask participants to internalize action principles) was not successful.

In regard to Hypothesis 1, we were not able to find a direct, beneficial effect of error management culture on team performance. As in Study 1, culture strength did not moderate the relationship between error management culture and performance (Hypothesis 2). However, in regard to Hypothesis 3, we found an indirect, beneficial effect through communication in that our manipulation that included action principles fostered perceived error management culture, which increased communication, and communication fostered team performance.^3^

## General Discussion

In the present paper, we investigated whether and how error management culture may be induced in newly formed teams, and if and how error management culture can be beneficial for performance, both in terms of rational and creative problem solving. We found that inducing error management culture is more difficult than expected. Our manipulation that included action principles that aimed to foster internalization was successful in terms of an effect on perceived error management culture. However, we did not find a direct effect of error management culture on performance, as it was previously found in field studies. This raises the question whether we actually succeeded in inducing error management culture, or whether our results rather reflect mere compliance with our instructions. In Study 2, where we included communication as a mediator, we found error management culture to be beneficial for performance indirectly via communication. In both samples, we further tested whether culture strength moderated the effect of error management culture on performance. We did not find the expected moderation effect. In the following, we will discuss our findings in greater detail, and suggest potential theoretical explanations.

### Theoretical Contributions

#### Inducing an Error Management Culture

To the best of our knowledge, the present studies are among the first ones to investigate how an error management culture can be induced. In two studies, we used manipulations that included action principles (Glaub et al., 2014) in regard to dealing with errors – “error principles.” We had explicitly asked participants to write down these principles and follow them throughout working on the task(s). Additionally, we repeated our manipulations several times, and had a “reminder” of the principles visible at all times during the team discussion. By repetition of the main principles of our manipulations, we aimed to foster that participants internalize these principles (Gal’perin, 1967). As in Study 2, our manipulation that did not include these “error principles” was not successful, including such principles seems to be important when inducing error management culture.

It has to be noted that while we succeeded in terms of an effect of our manipulation on perceived error management culture, we did not find an effect (neither of our manipulation, nor of perceived error management culture) on performance. An explanation may either lie in the error management culture and performance relationship, or in the culture strength regarding error management culture. In the following, we discuss both possibilities in greater detail.

#### The Error Management Culture–Performance Relationship

Contrary to what we had expected based on the literature on error management culture in organizations (e.g., van Dyck et al., 2005; Fischer et al., 2018), we did not find a direct effect of error management culture on performance, neither in terms of rational, nor in terms of creative problem solving. One potential interpretation could be that error management culture is not beneficial for performance. This, however, would be contrary to findings in, for example, the aforementioned field studies. Additionally, experimental evidence on the individual level has repeatedly demonstrated a beneficial effect of error management training on performance (e.g., Keith and Frese, 2005). While we were able to successfully induce error management culture in terms of an effect on perceived error management culture in both studies, only in Study 2, where we included communication as mediating variable, we found that error management culture had an *indirect* effect on performance in terms of creative problem solving through increased communication among team members.

On the one hand, we were surprised that we did not find the direct effect of error management culture on performance that has been reported non-experimental field studies (e.g., van Dyck et al., 2005; Fischer et al., 2018). On the other hand, there is a major difference between the organizations studied in non-experimental field studies and the teams in our studies: In organizations, the organizational culture is most likely engrained and internalized by the members of the organization. The teams in our study were newly formed and comprised of strangers who had no prior interaction. Consequently, the teams did not have an already internalized culture, thus had to adopt a new culture. It is possible that in such situations where culture has to be newly formed and unfold, error management culture may take more time to fully unfold, or may not be strong enough to directly impact performance – communication may be the key driver.

#### Culture Strength

Our second potential explanation to why we did not find an effect of error management culture on performance lies in the culture strength, i.e., the agreement about the groups’ culture between team members (Schneider et al., 2002). We thus tested whether culture strength moderates the relationship between perceived error management culture and performance. We did not find culture strength to moderate the relationship of error management culture and performance. In other words, the relationship between perceived error management culture and performance did not depend on team members’ agreement about the group’s culture. This result reinforces our assumption that we did not actually succeed in inducing culture, and the effect of our manipulation on error management culture rather represents participants’ superficial compliance with our instructions. It has to be noted that when controlling for the (lack of) agreement, perceived error management culture was related to increased team performance.

### Practical Contributions

An error management culture conveys a constructive view on errors as well as strategies for dealing with errors that have occurred. Negative error consequences, such as failure (Shepherd et al., 2011; Frese and Keith, 2015) shall be prevented, and positive consequences, such as learning from errors, shall be encouraged. Based on the previous findings that error management culture is beneficial for organizational performance, the question of how an error management culture can be induced is important for practitioners.

With our studies, we provide a starting point that outlines what interventions in change processes and mergers and acquisitions should consider and include. Our studies demonstrated that in order to induce error management culture, action principles in regard to errors shall be internalized, and thereby shape culture. These “error principles” may include rules how to deal with errors in the team or organization. For example, these “error principles” may explicitly encourage communicating an error that has occurred. By sharing the error with others – without having to fear blame or other negative consequences – other people may learn from the error. Furthermore, the error, once shared with others, may be used as a starting point to develop new, innovative ideas. Ultimately, this may enhance team or organizational performance.

### Strengths, Limitations, and Future Research

One of the strengths of our studies is that we used different sources for all our variables. In both our studies, our independent variable was an aggregate measure of the respective team members. In Study 1, our dependent variables were objective measures. In Study 2, the dependent variables were assessed and counted by raters, and communication was objectively measured. Thereby, we were able to circumvent the common source bias, which is a problem in many studies.

Furthermore, replication is essential to reduce the likelihood of false-positive findings. In abductive research, (internal) replication is “a viable antidote to what Bliese and Wang (2020) term ‘origination bias,’ or in other words, ‘the practice of viewing findings from a single, original study as being almost sacred,’ even if these findings were exploratory in nature” (Bamberger, 2019, p. 104). In Study 2, we were able to replicate the findings regarding our manipulation containing action principles we obtained in Study 1. Moreover, we were able to extend our model by including communication as well as an indirect effect on performance.

Nonetheless, some questions remain to be answered. First, culture may unfold its beneficial effects over time. The present studies have largely neglected a temporal perspective on error management culture. In the present studies, newly formed teams worked together for 30 to 60 min. This may not be long enough for a (team) culture to establish, or to unfold effects on team performance. Particularly for newly formed teams, as was the case in the present studies, this time frame may not be long enough to establish shared practices (e.g., House et al., 2004). Future studies should observe teams over a longer period of time. For example, when conducting studies with university students, student teams could be observed throughout one semester. One possibility would be to form groups of first semester students, as most likely, first semester students do not know each other yet. With these newly formed teams, research could test different versions of error management instructions that may focus on different aspects of error management and error management culture (e.g., error detection, error handling, error communication). Students could be randomly assigned to either one of these classes, or a “control class” that does not make explicit statements about how errors should be dealt with. Over the course of an entire semester, it could be observed how an error management culture unfolds, and whether the performance of students in the “error management classes” was better in comparison to the control condition. As field studies have demonstrated beneficial effects of error management culture, error management training has been shown to be beneficial for performance, and the “control classes” represent more or less every “typical” class at university nowadays, institutional review boards should approve an application for such a study. If students would agree to provide their student registration number, such a design would even allow to study long-term effects on performance in terms of the grade point average. Taking the temporal perspective into consideration is a promising area for future research.

Moreover, we consider it possible that the group size in our studies is too small for a team culture to establish. All the teams in the studies reported in the present paper were either dyads or triads. We particularly aimed at recruiting more than two people (whenever possible), as we assumed team dynamics to unfold in teams with at least three members. One key element of error management culture is communication. Usually, this encompasses error communication. In Study 2, we have demonstrated that the mere quantity of communication was associated both with error management culture *and* with performance. Based on the tentative finding that communication may be an important variable for the error management culture and performance relationship, the size of the teams in our studies (two or three team members) may not have been adequate. In teams that are comprised of more members, there may be more chances and instances of communication. This increased (possibility for) communication may reinforce the error management culture. This may be a hint that an error management culture may unfold more easily in teams comprised of more team members. Future studies should thus attempt to recruit teams that are comprised of more members.

In that sense, the group size may also explain our somewhat surprising finding that culture strength did *not* moderate the relationship between error management culture and performance. It is possible that in within-group agreement might be more difficult to achieve in small groups as compared to medium-sized groups. We thus suggest that in future research with teams comprised of three or more members, the moderating role of culture strength should be further investigated.

We believe that even considering the limitation of the relatively small group size in our sample, the merit of our manuscript is to provide first ideas of how to induce an error management culture. Therefore, for this purpose, we believe that including dyads (while statistically controlling for the group size) is justified. Future studies should, if possible, use groups of three or even more members as level of analysis. Based on our experience, we suggest that for practical reasons, such experiments should be conducted in a laboratory setting (as opposed to online). The current Covid-19-crisis seriously limits researchers’ options for the moment being.

Furthermore, it remains to be tested whether our intervention can be successfully applied in organizational settings, where teams typically have already been formed. It seems plausible that interventions would have to be even stronger in order to “overrule” already established norms and practices regarding errors in such teams or organizations. Second, future research could explore how sustainable, i.e., long-lasting, the effects of such interventions are. Answering this question could be particularly important in view of change processes and mergers and acquisitions. For example, for how long should interventions regarding a cultural integration of the companies in a merger and acquisition be in effect? Many organizations would benefit from answers to such questions.

Admittedly, in hindsight, the tasks themselves may not have been optimal to study the phenomenon, because it may not have been obvious to the participants right away whether they had made an error, or what the error was (we further discuss this issue in the following paragraphs). In fact, this may be quite realistic, as many employees’ tasks may not provide immediate feedback. We regard the *variety* of tasks we employed as strength. In Study 1, we employed tasks that require rational problem solving, and where the degree of correctness of the teams’ solution can be judged objectively. In Study 2, participants worked on an actual task that requires creative problem solving: we had asked the teams to create a marketing plan for a given product. The task is a simulation of an actual work task. In fact, participants in Study 2 were online freelancers (“eLancers”), who believed to be working on an actual task for an actual company. This allowed us to combine advantages of an experimental setting, such as standardization, with advantages of field studies, such as task engagement. This contributes to a high applicability and generalizability of our results.

The task we used in Study 2 requires creativity, and may be quite similar to a brainstorming task. This procedure may raise the question of what actually constituted an error in the task. It is true that in the early stages of a brainstorming task, all ideas shall be voiced, regardless of whether they can be implemented or not. However, participants of Study 2 were not asked to provide as many creative ideas as possible; rather, they were asked to create a marketing plan for a (seemingly) real company and a (seemingly) real product. As such, ideas that were generated in an early phase of working on the task had then to be evaluated by the groups regarding their applicability in “real-life.” Admittedly, in the process of brainstorming, creative ideas that may not be applicable in real-life settings (which we would consider “erroneous”) may lead to good ideas that can be implemented. We do not consider this a contradiction to our concept of error management; Quite the opposite: embracing these “erroneous” ideas that the group rejects in the process of their discussion and acknowledging their potential may result in innovative yet applicable results.

On a similar notion, in Study 1, the errors the group made are represented by the deviations of their solution to the expert solution. The first stage of the task is to individually rank the items. In the following stage, the group discussion, team members often first analyze their individual rankings, and then “negotiate” a common solution. During this discussion, team members often explain their reasoning when ranking the items. Thereby, (individual) errors may become obvious and be discussed. For example, in the Landing on the Moon task, some participants correctly explained that matches do not work on the moon, as there is no oxygen that would light them up. In the Survival at Sea task, some participants realize that mosquito nets are not required on the ocean, as there are no mosquitos. During the group discussion, these insights may help overcoming individual errors when negotiating a common solution.

Research that has assessed innovator resilience potential (Todt et al., 2018), which includes “state-like qualities that are essential prerequisites for innovative functioning and coping with future setbacks (Todt et al., 2018, p. 522), demonstrated that effects are particularly strong after having experienced an innovation project termination before. For the context of our studies, one could conclude that the error management and performance relationship may be particularly strong when groups have experienced errors. While this idea definitely has its merits, we believe that there are several points that need to be considered: First, we agree that in order to observe specific behavior toward errors, errors actually need to take place. For example, in our first study, errors were operationalized as the deviations from the expert solutions to the Landing on the Moon and Survival at Sea task. As none of the groups were able to find the correct solution, we could say that they all made errors in the process of finding their solution. We are thus not able to directly compare groups that made no errors at all with groups that made errors. From a theoretical perspective, the concept of error management culture goes above and beyond behavioral reactions to errors (e.g., to discuss the error with a colleague); it can be described as a “mind-set” toward errors. The mind-set includes attitudes toward errors (e.g., to consider them as learning opportunities) or emotions toward errors (e.g., the absence of error strain). These do not require actual errors to happen.

As Study 2 was conducted online, we have little control over the situation in which participants took part in the study (for example, were participants distracted, etc.). Therefore, we took careful measures to enhance task engagement (fair compensation, attention checks, statistically controlling for goal commitment, using a real task). For studying a team concept, an online environment may be not be optimal, as team members do not even see each other – in the studies we had conducted, team members merely exchanged text messages. Again, as the pattern of results is similar to that of Study 1, the environment in which we conducted the study seems to have not affected our results.

## Conclusion

A vast body of field studies has demonstrated the beneficial effect of error management culture on performance. In the present studies, we aimed at exploring the error management culture and performance relationship in an experimental setting. In two experiments with teams, we discovered that inducing an error management culture is more difficult than expected, and that the relationship is not as clear as expected. While we found a beneficial, yet indirect, effect via communication on performance in terms of creative problem solving, we were not able to find a direct effect on performance in terms of rational problem solving. One important question that remains unanswered is the temporal perspective. We encourage future research to further look into boundary conditions on the error management culture and performance relationship: employing different tasks may conclude in different results. Still, our studies are an important starting point in gaining a better understanding of the relationship between error management culture and performance.

## Data Availability Statement

The datasets of the studies are available from the authors on reasonable request.

## Ethics Statement

The studies involving human participants were reviewed and approved by Leuphana University of Lüneburg. The patients/participants provided their written informed consent to participate in this study.

## Author Contributions

All authors developed the theoretical ideas and study design. AK and DH collected the data, performed analyses, contributed to the method and results sections, and wrote the manuscript. NK and MF provided critical revisions.

## Conflict of Interest

The authors declare that the research was conducted in the absence of any commercial or financial relationships that could be construed as a potential conflict of interest.

## Publisher’s Note

All claims expressed in this article are solely those of the authors and do not necessarily represent those of their affiliated organizations, or those of the publisher, the editors and the reviewers. Any product that may be evaluated in this article, or claim that may be made by its manufacturer, is not guaranteed or endorsed by the publisher.

## Footnotes

1 We are aware of the debate going on whether the definition is of culture or of climate (e.g., Reichers and Schneider, 1990; Denison, 1996). We follow Fischer et al. (2018) and do not take part in this debate; rather, we keep the term *error management culture* that has been introduced by van Dyck et al. (2005).

2 Calculations for the demographics is based on *N* = 303, as six participants did not respond to demographic questions.

3 Note that the descriptive statistics provided in Table 6 reveal that while groups in the Error Management framing condition scored higher on perceived error management culture, groups in the “slimmed” Error Management framing condition scored higher on communication and quality and quantity of the ideas. (Note that these results are only a tentative description of the descriptive statistics).
